# Imaging grading system for the diagnosis of dural ossification based on 102 segments of TOLF CT bone-window data

**DOI:** 10.1038/s41598-017-03178-x

**Published:** 2017-06-07

**Authors:** Sheng-yuan Zhou, Bo Yuan, Xiong-sheng Chen, Xue-bin Li, Wei Zhu, Lian-shun Jia

**Affiliations:** Department of Orthopaedics, Shanghai Changzheng Hospital, Fengyang Road No. 415, Shanghai, 200003 China

## Abstract

Thoracic ossification of the ligamentum flavum (TOLF) complicated with dural ossification (DO) is a severe clinical disease. The diagnosis of DO preoperatively remains challenging. The current study retrospectively analyzed imaging features of 102 segments with TOLF from 39 patients and proposed a grading system for evaluating DO risk. Logistic regression results showed that unilateral spinal canal occupational rate (UCOR), tram track signs, and C-signs were all risk factors for DO (odds ratios of 5.393, 19.734 and 72.594, respectively). In validation analyses for the TOLF-DO grading system, sensitivity was 76.0% (19/25), specificity was 91.0% (70/77), and Youden’s index was 0.66. Thus, implementation of the TOLF-DO grading system has the potential to improve the diagnosis of DO.

## Introduction

Thoracic ossification of the ligamentum flavum (TOLF) complicated with dural ossification (DO) is an uncommon but severe clinical entity^1–4^. The pathogenesis of DO remains unclear at present. The diagnosis of DO usually depends on the finding of an “ossified mass” unseparated from the dural sac during operation. Surgical treatment for TOLF-DO is more difficult and risky than simple TOLF^5–9^. If the presence of DO can be confirmed preoperatively, the safety of the surgical procedure can be ensured to a greater extent. However, it is still difficult to confirm the diagnosis of DO preoperatively based on patient history, clinical observation, and imaging presentation.

Imaging research regarding TOLF-DO is relatively rare. Muthukumar *et al*.^10^ and Sun *et al*.^11^ reported that the presence of a “tram track sign” (i.e., a hyperdense bony excrescence with a hypodense center) or “comma sign” (i.e., evidence of ossification of one-half of the circumference of the dura mater) on transverse computed tomography (CT) or magnetic resonance imaging (MRI) scans was significant for the diagnosis of DO. In retrospective case data, both tram track signs and comma signs could be seen in the imaging of TOLF-DO patients. In clinical practice, the ossified mass of the TOLF-DO segment is frequently hypertrophied, while the less hypertrophic adjacent segments are usually not complicated with DO. This pattern suggests that the degree of hypertrophy in the ossified mass may be associated with the occurrence of DO.

In the present study, we retrospectively analyzed imaging features of the segments definitely affected by TOLF-DO and compared them to those of TOLF patients without DO. Based on the statistical results, we propose a grading system for evaluating DO risk and offer this system as a practical tool for the diagnosis of TOLF-DO.

## Results

### Demographic and clinical characteristics

The study sample included a total of 102 segments with TOLF from 39 patients. Twenty-five of these segments were diagnosed with TOLF-DO, and the other 77 segments were diagnosed as TOLF without DO (TOLF-NDO). The morphological features of the involved segments were observed on bone-window CT transverse images. DO segment locations are listed in Table 1.

**Table 1:** Segments locations of patients with TOLF.

	TOLF-DO	TOLF-NDO
C_7_/T_1_	0	1
T_1/2_	0	4
T_2/3_	1	6
T_3/4_	1	5
T_4/5_	0	5
T_5/6_	2	4
T_6/7_	2	2
T_7/8_	1	3
T_8/9_	2	3
T_9/10_	6	12
T_10/11_	5	20
T_11/12_	5	9
T_12_/L_1_	0	3

The percentage of patients with TOLF-DO in our series of patients was 41.0% (16/39), and the percentage of segments affected by TOLF-DO was 24.5% (25/102). T9–T12 were the segments most commonly affected by DO, accounting for 64% of DO cases (16/25).

### TOLF-DO Grading system

**Figure 1:**
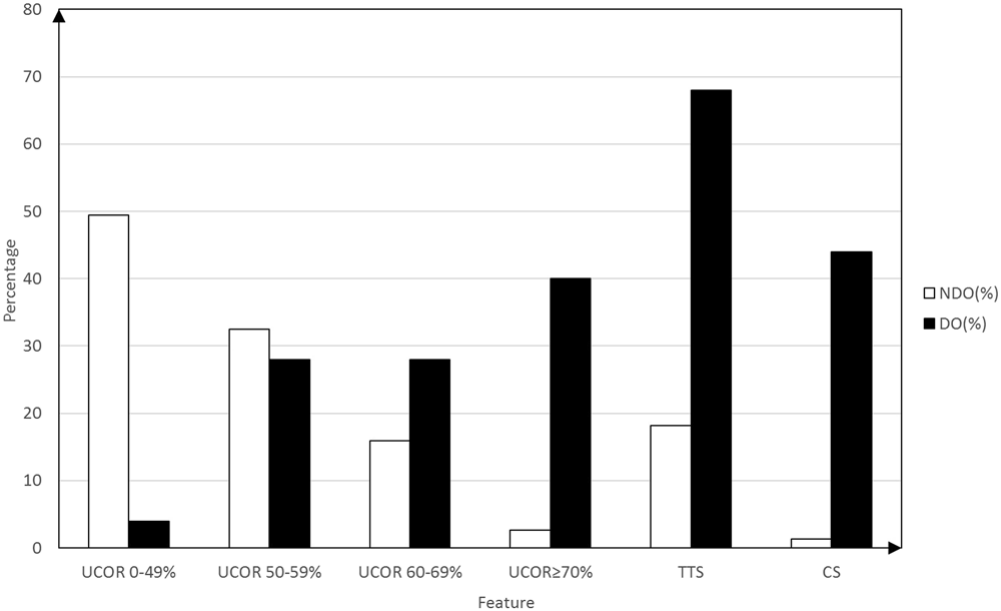
Distribution of UCOR, tram track sign and C-sign values. UCOR indicates unilateral spinal canal occupational rate. TTS indicates the tram track sign. CS indicates the C-sign. NDO indicates TOLF without DO. DO indicates TOLF with DO.

We divided unilateral spinal canal occupational rate (UCOR) measurements for 102 TOLF segments into four ranges (0–50%, 50–59%, 60–69%, and 70–100%). The incidence of DO increased gradually with increases in UCOR values. For UCOR values ≥ 60%, the incidence rate of TOLF-DO was higher than that of TOLF-NDO. Tram track sign and C-sign rates in DO segments were also higher than in NDO segments (Fig. 1).

Logistic regression results indicated that UCOR, tram track sign and C-sign were all risk factors for DO (Table 2). Based on odds ratios calculated from this model, we designed a TOLF-DO grading system (Table 3). The system comprised 4 total points, and the existence of DO was highly suspected when the score was greater than 2 points.

**Table 2:** Odds ratio of TTS, CS and UCOR ≥ 60%.

Variable	Odds ratio with 95% CI	P value
TTS	19.734 (3.364, 115.758)	0.001
CS	72.594 (5.467, 963.961)	0.001
UCOR ≥ 60%	5.393 (1.021, 28.468)	0.047

**Table 3:** TOLF-DO grading system.

Parameter	Score
**Unilateral COR value**	
<60%	0
≥60%	1
**Radiological features**	
Tram track sign	1
C-sign	2
Tram track sign + C-sign	3

The existence of DO was highly suspected when the score was larger than 2 points.

### Clinical validation of the TOLF-DO grading system

**Figure 2:**
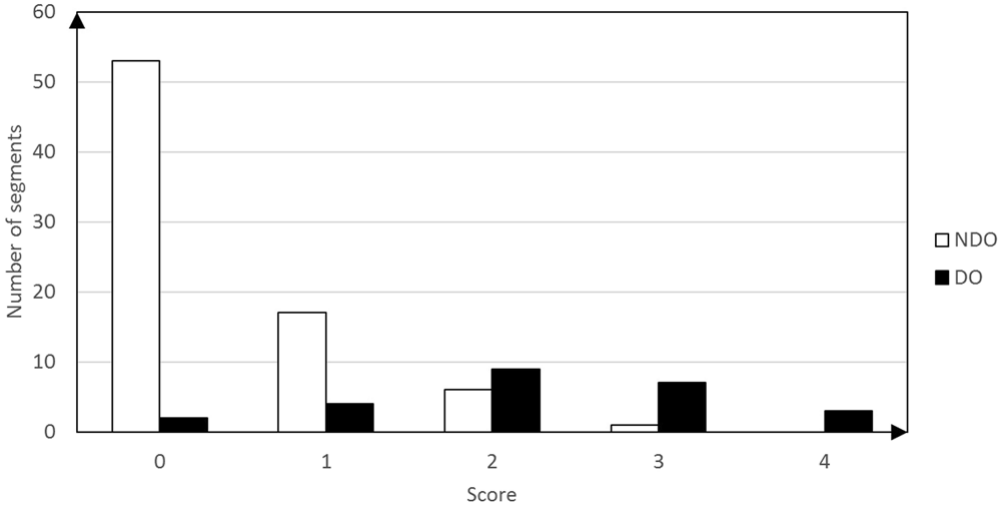
Results of the TOLF-DO grading system and score distribution for the 102 segments.

Figure 2 displays the distribution of TOLF-DO risk scores across the 102 segments. Table 2 displays the results of the logistic regression analysis. Tram track sign, C-sign and UCOR ≥ 60% were all risk factors for DO. Table 4 lists diagnostic outcomes for the TOLF-DO grading system and intraoperative diagnosis.

**Table 4:** The comparison of diagnostic outcomes in TOLF-DO grading system and Intraoperative diagnosis.

		TOLF-DO grading system	
		+	−
Intraoperative diagnosis	+	19	6
	−	7	70

Sensitivity = 19/25 * 100% = 76.0%. Specificity = 70/77 * 100% = 91.0%. Positive predictive value = 19/26 * 100% = 73.1%. Negative predictive value = 70/76 * 100% = 92.1%. Youden’s index = 0.76 + 0.90 − 1 = 0.66. Crude agreement rate = (19 + 70)/(19 + 6 + 7 + 70) * 100% = 87.3%.

## Discussion

TOLF-DO is not uncommon in TOLF. Clinical data in the current study suggested that the incidence of TOLF-DO was 41.0%, compared to estimates of 14% to 61% in previous literature^2–4, 12, 13^. DO often occurs at lower thoracic segments, and the affected region was located at T9–12 for 16 of the 25 segments in our series. Other studies have also reported DO most frequently at T9–12. This region is also the preferred site for TOLF^14–16^.

We retrospectively reviewed CT bone window imaging data for 102 TOLF segments. “Comma signs” had more diagnostic value than “tram track signs” for TOLF-DO. The tram track sign was observed in 17 segments, but the “C-sign” was observed in only one TOLF-NDO segment. The relationship between tram track signs and DO thus requires further clarification. Some researchers have found that a layered presentation (i.e., double-layer sign) in the ossified mass of the posterior longitudinal ligament (OPLL) or a C-shaped (C-sign) high-density ossified zone on the side of the dura is valuable for the diagnosis of DO^17–19^. The low-density zone in the double-layer sign has been explained as an immature ossified ligament^20, 21^ in the ossified mass. The double-layer sign also indicates that OPLL originates from the dura-side ligament, which increases the possibility that it is accompanied by DO^16, 18, 22^. Although tram track sign is not a unique imaging feature of TOLF-DO, the existence of DO should be highly suspected in cases where the C-sign is observed. Accordingly, the proposed grading system highlights the diagnostic value of C-sign in DO.

The incidence of DO is apparently correlated with the progress of TOLF^23^. DO was not observed when the UCOR value was lower than 40%; in contrast, the risk of DO increased significantly when UCOR was at least 60%. To date, no studies have reported a correlation between the occurrence of DO and the thickness of the ossified mass. More TOLF patients are needed to validate the diagnostic significance of TOLF-DO. There is no uniform method to measure COR values in TOLF. The simple and practical measurement of UCOR values for TOLF that we recommend in this study was based on the origin and progression of OLF.

The TOLF-DO grading system had a high specificity (91%) for the diagnosis of DO. For scores less than 2 points, the existence of DO was not suspected. In contrast, the sensitivity was 76%, indicating a 24% rate of missed DO diagnoses. According to the distribution of UCOR values, 10 segments in the 12 cases with UCOR ≥ 70% were diagnosed with DO. We did not evaluate the grading system for UCOR values ≥ 70% because the number of cases meeting this criterion was relatively small. However, according to our clinical experience, the presence of DO should be highly suspected when UCOR values exceed 70%.

The grading system setting and clinical validation were both based on a relatively small number of cases. The feasibility and significance of applying this system to the diagnosis of DO require further investigation with larger sample sizes and in multicenter clinical studies.

## Methods

### Ethics statement

The treatment protocol and informed consent were approved by the Shanghai Changzheng Hospital Institutional Review Board, and all subjects gave informed consent. All methods were performed in accordance with the Declaration of Helsinki.

### CT Imaging examination

#### Tram track sign and C-sign

**Figure 3:**
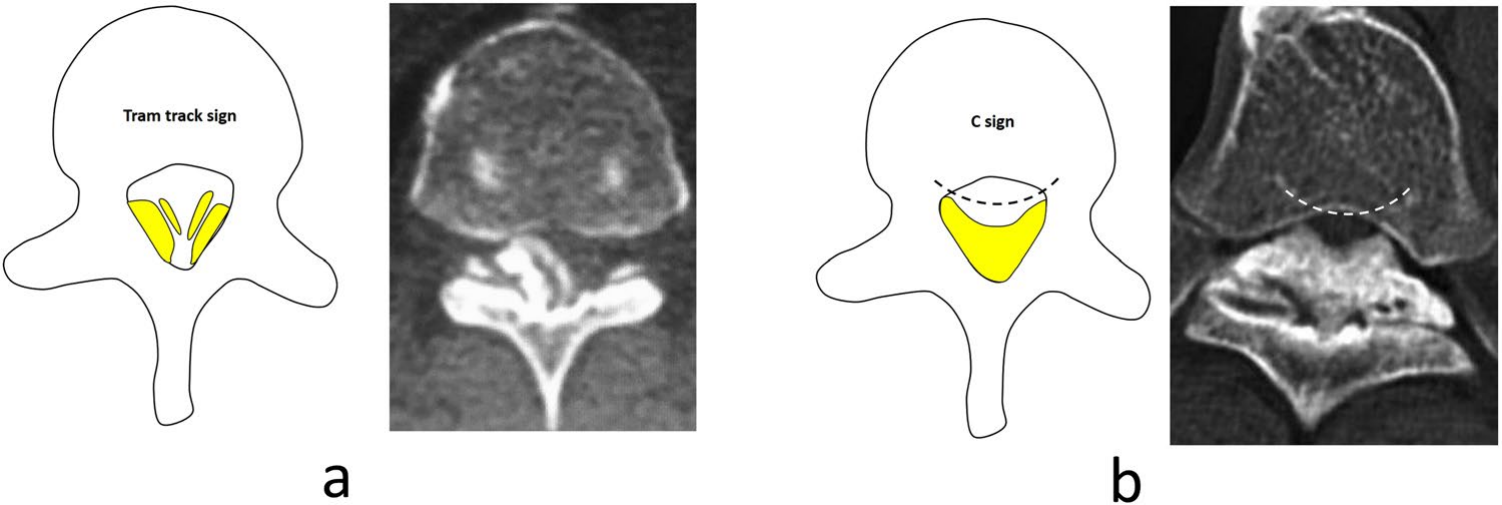
(**a**) Schematic diagram (left) and CT transverse scan (right) of the tram track sign; (**b**) Schematic diagram (left) and CT transverse scan (right) of the C-sign.

The TTS is a hyperdense bony excrescence with a hypodense center (Fig. 3a)^10^. The edges of the bilateral dura-side ossified mass are curved, forming a “C” sign (Fig. 3b). The C-sign includes all the manifestations of the comma sign^10^.

#### Measurement of unilateral spinal canal occupational rate (UCOR) for the ossified mass

**Figure 4:**
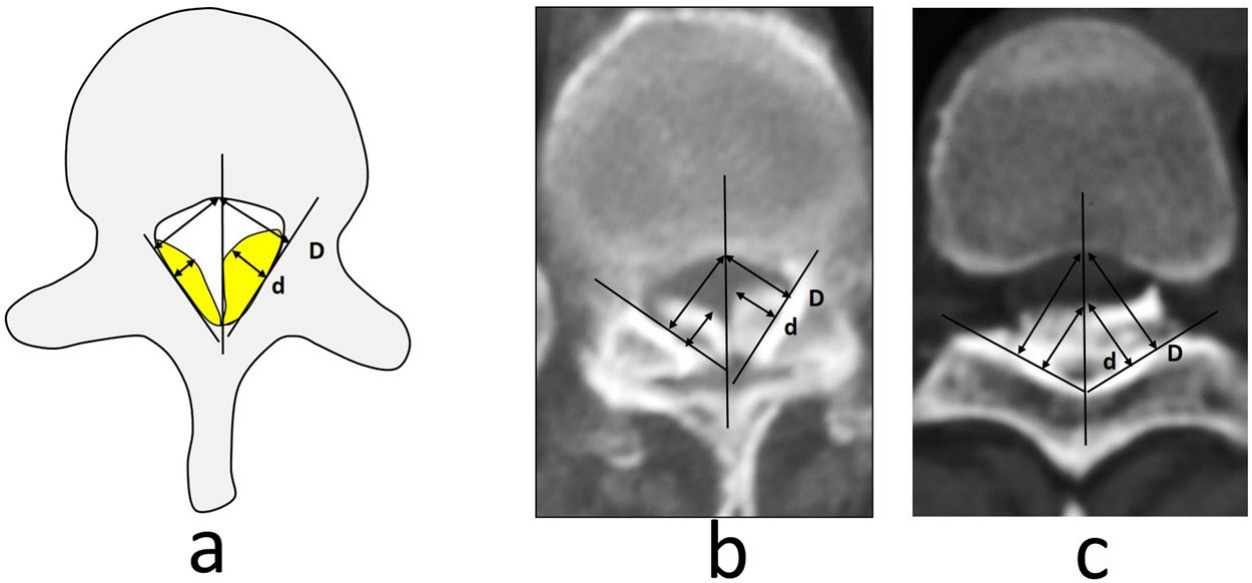
UCOR measurements for the ossified mass, where UCOR = d/D × 100%. (**a**) Schematic diagram of the measuring method. (**b**) Measurement on CT transverse scan through facet joint. (**c**) Measurement on CT transverse scan through vertebral lamina.

OR severity was measured on CT transverse images. The ossified mass of the OLF originates from the bilateral laminae and facet joints, and it then spreads to the midline and posterior vertebral wall. The maximum thickness of the unilateral ossified mass (d) and the distance from the lamina to the midpoint of the posterior vertebral wall (D) were measured. The ratio of d/D was calculated, and the larger value was set as the UCOR (Fig. 4).

#### Establishment and clinical validation of the TOLF-DO Grading system

Logistic regression was used to evaluate risk factors for TOLF-DO. According to odds ratios for multiple risk factors, we established a TOLF-DO grading system for evaluating risk for DO.

The 102 TOLF segments were evaluated using the TOLF-DO grading system. Intraoperative diagnosis was used as the gold standard. The sensitivity and specificity of the grading system for diagnosis of TOLF-DO were calculated.

### Statistical Analysis

Multivariate analyses of risk factors for TOLF-DO were carried out with logistic regression to determine odds ratios (OR) and their 95% confidence intervals (95% CI). A two-tailed P value less than 0.05 was regarded as statistically significant. All statistical analyses were performed using SPSS (version 18.0, SPSS Inc., Chicago, IL).
